# Making predictions in a changing world—inference, uncertainty, and learning

**DOI:** 10.3389/fnins.2013.00105

**Published:** 2013-06-14

**Authors:** Jill X. O'Reilly

**Affiliations:** Nuffield Department of Clinical Neurosciences, FMRIB Centre, John Radcliffe Hospital, Oxford UniversityOxford, UK

**Keywords:** change detection, uncertainty, exploratory behavior, modeling, bayes theorem, learning

## Abstract

To function effectively, brains need to make predictions about their environment based on past experience, i.e., they need to learn about their environment. The algorithms by which learning occurs are of interest to neuroscientists, both in their own right (because they exist in the brain) and as a tool to model participants' incomplete knowledge of task parameters and hence, to better understand their behavior. This review focusses on a particular challenge for learning algorithms—how to match the rate at which they learn to the rate of change in the environment, so that they use as much observed data as possible whilst disregarding irrelevant, old observations. To do this algorithms must evaluate whether the environment is changing. We discuss the concepts of likelihood, priors and transition functions, and how these relate to change detection. We review expected and estimation uncertainty, and how these relate to change detection and learning rate. Finally, we consider the neural correlates of uncertainty and learning. We argue that the neural correlates of uncertainty bear a resemblance to neural systems that are active when agents actively explore their environments, suggesting that the mechanisms by which the rate of learning is set may be subject to top down control (in circumstances when agents actively seek new information) as well as bottom up control (by observations that imply change in the environment).

To function efficiently in their environment, agents (humans and animals) need to make predictions. We can think of predictions being based on an internal model of the environment, stored in the brain, which represents information that has been observed, and predicts what will happen in future. The process by which such a model is constructed and updated may be called a learning algorithm. Learning algorithms are of interest to neuroscientists, partly because such algorithms actually exist in the brain (and we would like to understand them) and partly because constructing learning algorithms that model participants' incomplete knowledge of task contingencies can help us to understand their behavior in experimental paradigms.

Whilst all knowledge of the environment is arguably acquired through learning, learning is particularly important in environments that change over time. In this review we are concerned with a particular computational problem that arises in complex changing environments—how should learning algorithms adapt their learning rate to match the rate of change of the environment. We will consider two key concepts in inferring the rate of change: the likelihood function, by which the likelihood that current and past observations were drawn from the same distribution is evaluated, and the prior probability of change, which constrains how much evidence will be required for the learning algorithm to infer that a change has in fact occurred. We will relate these two constructs to the concepts of expected and estimation uncertainty, and consider the interplay between uncertainty and learning. Finally we will consider neural correlates of uncertainty and learning, and ask whether these are the same when learning is driven bottom up by surprising observations, and top down as part of the process of actively exploring the environment.

## Why is change a challenge for learning algorithms?

A learning algorithm is an algorithm that makes use of past experience to construct a representation of the learned-about subject (we will call the learned-about subject “the environment” in this article). The purpose of learning is to predict future observations of the environment and hence respond to them efficiently (Friston and Kiebel, 2009; Friston, 2010). Therefore, to function effectively it is essential that the representation developed by the learning algorithm accurately reflects the *current* state of the environment and/or is predictive of *future* environmental states.

Throughout this review, when I mention a changing environment, I mean an environment that changes to an unknown state. Environments can change in both predictable and unpredictable ways. A *predictably changing* environment would be a changing environment whose state can nevertheless be predicted precisely as a function of time—for example, the phases of the moon. An *unpredictably changing* environment could be defined as an environment that undergoes changes that move it to an unknown state. For example, the location of the TV remote control in a family living room often behaves like this. In terms of this discussion of learning algorithms, we are only really interested in the second type of change—in the first case (an environment which changes, but predictably) there is nothing new to learn.

## The key challenge: how far back should you look?

Given that the changing environment is not totally random over time (in which case learning would be useless), a learning algorithm can make use of a history of data extending beyond the most recently experienced observations, to inform its internal representation of the environment. The more past data that can be validly used to create a representation of the environment, the more accurate the representation is likely to be. However, “validly” is the key word because in a changing environment, the challenge is to decide exactly which data should be used to create an up-to-date representation, and which data are no longer relevant (Doya, 2002; Behrens et al., 2007).

To illustrate the point: in a stationary environment (an environment which does not change over time), all data from the past, no matter how old, could be used to inform an internal representation of the current state of the environment. Therefore, for example, in a stationary environment, the mean of *all* observations would give the most accurate estimate possible of the mean of the underlying distribution (the environment) from which future observations will be drawn.

In contrast, in a changing (non-stationary) environment, it is not true that the distribution of all past observations reflects the underlying distribution in force at any particular time point *i*. On the contrary, in a changing environment there is a need for an additional layer of processing to work out how observations from different times in the past predict future states of the environment. For example, if the environment has undergone an abrupt change, the best solution may be to identify the change point and use all data since that point, disregarding data from prior to the change point. There is a trade-off between using as much data as possible (to increase the accuracy of the representation) and leaving out old data, which may be irrelevant or misleading.

### A simple way to discount older data: decay kernels

**Figure 1:**
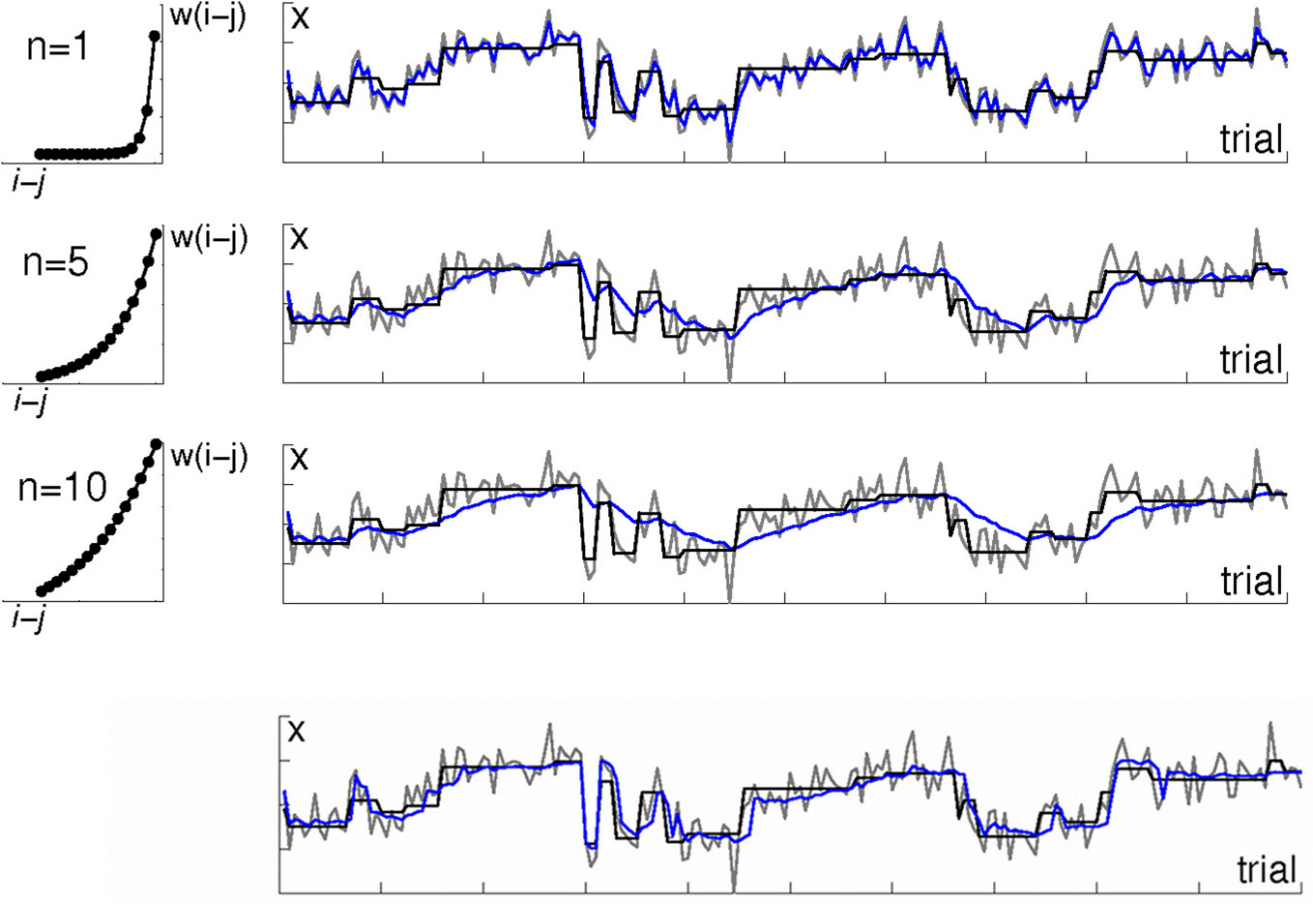
**Algorithms with a fixed temporal discount do not fit well to environments with a variable rate of change**. The right-hand panels illustrate an environment in which observations are drawn from a Gaussian distribution; each row shows a different learning algorithm's estimate of the distribution mean μ. The mean μ, which has period of stability interspersed with sudden change, is shown in black. Actual observations *x* are shown in gray. Estimates of μ are shown in blue. The top three rows are kernel-based learning algorithms with different time constants. The left hand panels illustrate the three weighting functions (kernels) which were used to determine the weighting of observations in the panels next to them. The weighting w(*j*) assigned to observation *i* − *j* when calculating the mean μ (*i*) on observation *i* is defined by the exponential function *w*(*i*) = exp(−*j/n*). The rate of decay is determined by the constant *n*, with higher values of *n* meaning a longer period of the past is used. The top row shows a kernel using only very recent observations. This tracks the mean μ well, but jumps around a lot with individual observations. Note the blue line tracks the gray (data) line more closely than it tracks the actual mean μ (black line). The 2nd and 3rd rows show kernels using longer periods of the past. This gives a much smoother estimate, but is slow to adjust to changes in μ. The bottom row shows the output of a Bayesian learning algorithm that includes an additional level of processing in order to detect change points. Note how unlike the kernel-based algorithms, its estimate is stable during periods of stability *and* changes rapidly in response to change in the underlying distribution.

Firstly, to illustrate the problems associated with adjusting to the rate of change of the environment, we will consider a simple but non-adaptive strategy for discounting old data: namely to discount or down-weight older observations. For example, an estimate of the mean of the underlying distribution at time point *i* could be based on a running average of the last *n* observations (*i* − *n*: *i*), or a kernel-based average where observations (*i* − *n*: *i*) are averaged using a weighting function which down-weights older observations (see Figure 1, left hand panels).

This simple, fixed kernel approach is easy to implement in data analysis, and one can imagine how it could be implemented simply in a neural network: Incoming observations each activate a set of neural nodes which represent them (for example, in a spatial map, nodes with spatial receptive fields in which stimuli appear would be activated by these stimuli); activation in the nodes decays gradually over time so more recently activated nodes contribute more to the total activity within the system, as in a “leaky accumulator” model (Usher and McClelland, 2001). This can be achieved using a single-layer neural network (Bogacz et al., 2006).

However, algorithms like the kernel-based approach just described that have a fixed rate of discounting old data rather than adjusting their parameters dynamically to account for periods of faster and slower change, perform poorly in environments in which the relevance of old data does not decay as a simple function of time (Figure 1). If the environment has periods of more- and less-rapid change, the ideal solution is to adjust the range of data that are used to inform the model over time, in accordance with how far into the past data are still relevant.

As an extreme example, consider an environment that has periods of stationarity interspersed with sudden changes (as in Figure 1). An algorithm that discounts older observations based solely on their age, like the simple fixed kernels described above, applies the same down-weighting to a past observation *i* − *n* regardless of whether a change has occurred since that observation, or not. If in fact a change has occurred since *i* − *n*, then the best solution would be to treat observations from before the change differently from those made since the change. On the other hand, during periods of stability, the best solution would be to use as many old observations as possible, not to arbitrarily disregard observations on the basis of age.

To implement a solution in which the range of data adjusts to changes in the rate of change of the environment over time, a learning algorithm would need some mechanisms by which to evaluate the rate of change of the environment. How can this be achieved?

## Estimating the probability of change

Consider a clear case in which not all past data are equally relevant—an environment which undergoes abrupt changes, interspersed with periods of stationarity (periods without change) as in Figure 1. How can a learning algorithm effectively disregard observations from before an abrupt change, whist using as much data as possible during stable periods? To do this, the learning algorithm needs to be able to infer the rate of change of the environment from the data it observes (Courville et al., 2006; Behrens et al., 2007; Wilson et al., 2010; Wilson and Niv, 2011).

In order to determine the rate of change of the environment, a learning algorithm needs to balance two considerations. Firstly, how unlikely was it that current observations were drawn from the same distribution (the same state of the environment) as previous observations? Secondly, how likely are change points themselves?—If I thought change points occurred on average about every 10,000 trials, I would need more evidence to infer a change than if I thought change points occurred on average every 10 trials (Wilson et al., 2010). We will now consider how these two considerations can be formalized.

### Inferring change I: the likelihood function

Let's start with the first of our two considerations: How unlikely was it that a given observation was drawn from the same distribution as previous observations? Consider a very simple learning task in which on each trial *i*, a target appears at some location across space, *x*_*i*_. The location is drawn from a Gaussian distribution with mean μ and variance σ^2^, such that *x*_*i*_ ~ 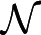 (μ, σ^2^).

Now let's say we observe a data point *x*_*i*_, and we want to know from what distribution this data point was drawn. In particular, we want to know whether this data point *x*_*i*_ was drawn from the same distribution as previous data points, or whether a change in the environment has occurred, such that the current parameters μ_*i*_, σ^2^_*i*_ are not equal to previous parameters from some putative pre-change point, μ_*i−n*_, σ^2^_*i−n*_.

Statisticians would talk about this problem in terms of *probability* and *likelihood*. We can calculate the *probability* that a certain observation (value of *x*_*i*_) would occur, given some generative distribution *x*_*i*_ ~ 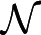 (μ, σ^2^), where the value of the parameters μ, σ^2^ are specified (for example, the probability of observing a value of *x*_*i*_ > 3 given that μ = 0 and σ^2^ = 1 is obtained from the standard probability density function for the Normal distribution, as *p* = 0.001). Conversely, we can think about the *likelihood* that the underlying distribution has certain parameters (the likelihood that μ, σ^2^ take certain values), given that we have observed a certain value of *x*_*i*_. The likelihood of some values of μ, σ^2^ given observations *x* can be written as *p*(μ, σ^2^|*x*_*i*_); conversely the probability of some observation *x* given certain parameters of the environment μ, σ^2^ can be written *p*(*x*_*i*_|μ, σ^2^). The two quantities are closely related:

(1) $$p(\text{μ},\sigma^{2}|x_{i})=p(x_{i}|\text{μ},\sigma^{2})$$

This relationship gives us a clear way to evaluate whether a change point has occurred—given some hypothesis about the parameters of the environment μ, σ^2^ that were in force prior to a putative change point, we can calculate the probability that an observation or set of observations made after the putative change point would have been observed given the pre-putative-change parameters of the environment, and hence calculate the likelihood that the pre-change parameters are in fact still in force (or conversely, the likelihood a change point has occurred).

It is worth noting that the likelihood function *p*(μ, σ^2^|*x*_*i*_), or more generally *p*(*parameters*/*observations*) can only be obtained in this way if the shape of the distribution from which observations are drawn is specified—we cannot estimate the parameters of a distribution, if we do not know how that distribution is parameterized. The validity pre-specifying the form of the generative distribution has been debated extensively throughout the twentieth century (McGrayne, 2011) and we will not rehash that debate here—we will simply note that whilst a wrong choice of distribution could lead to incorrect inferences, in practice it is often possible to make an informed guess about the distribution from which data are drawn—partly by applying prior experience with similar systems, and partly because types of observations follow certain distributions, for example, binary events can often be modeled using a binomial distribution.

### Inferring change II: prior probability of change and the transition function

Now let's address the second consideration for algorithms that adapt to the rate of change of the environment: the question of how likely change points themselves are, and the probability a-priori of particular transitions in the parameters of the environment.

We have already noted that, intuitively, an observer who believes change is improbable a-priori (for example, if the observer thinks that a change occurs only every 10,000 observations) should demand a higher level of evidence in order to conclude that a change has occurred, compared to an observer who believes change is frequent in his environment (e.g., if the observer thinks the environment changes about once every 10 trials). Furthermore, different environments can change in different ways over time—for example, in some environments the parameters might change smoothly, whilst other environments might change abruptly.

A function that models how the state of the environment evolves over time is called the *transition function* (Courville et al., 2006). A transition function defines how the state of the environment on trial *i* depends on its state on previous trials—so in the Gaussian example, the transition function specifies how the true parameters of the environment on trial *i* that is μ_*i*_, σ^2^_*i*_, depend on the true parameters of the environment on previous trials, μ_1:*i* − 1_, σ^2^_1:*i* − 1_.

Different transition functions represent different models of how the environment changes over time. For example, we could specify that the parameters of the environment vary smoothly over time, such that μ_*i*_ = μ_*i* − 1_ + δμ where δμ is small compared to μ. Alternatively, we could allow the parameters of the environment to jump to totally new values after a change point, for example by specifying:

(2) $$\left\{\mu_{i},\sigma_{i}^{2}\right\}=\{\begin{array}{cc} \left\{\mu_{i-1},\sigma_{i-1}^{2}\right\} & \text{if }J=1\\ \text{random} & \text{if }J=0 \end{array}$$…

where *J* is a binary variable determining the probability of a change, e.g., *J* follows a binomial B(0.1,1), giving a probability of 0.1 of a change on any given observation.

Both the form of the transition function (e.g., smooth change vs. jumps) and its parameters (e.g., the probability of a jump or the rate of smooth transition) are used to evaluate whether a change in the environment has occurred—models with transition functions specifying faster rates of change or higher probabilities of jumps in the parameters of the environment should infer change more readily than models that have low a-priori expectations of change.

### Bayes' theorem and change detection

We have seen that for a learning algorithm to adapt to the rate of change in the needs to evaluate the both likelihood of different states or parameters of the environment given the data, and the probability of change points themselves. These two elements are captured elegantly in Bayes' rule, which in this case can be written:

(3) $$p(\theta_{i}|x_{1:i})\propto p(x_{i}|\theta_{i})p(\theta_{i})$$…

where θ_*i*_ represents the parameters of the environment on the current trial *i*(μ_*i*_, σ^2^_*i*_) in our Gaussian example, and *x*_1:*i*_ are the observations on all trials up to and including the present one.

On the right hand side, *p*(*x*_*i*_|θ_*i*_) is equal to *p*(θ_*i*_|*x*_*i*_), the likelihood function, due to Equation 1 above; *p*(θ_*i*_), the prior probability of the parameters θ_*i*_, can be thought of as *p*(θ_*i*_|*x*_1:*i* − 1_) and is obtained from the estimate of the parameters of the environment on trial *i* − 1 via the transition function. For example if we model a transition function as in Equation 2, so that the parameters of the environment mostly stay the same from one trial to the next but can jump to totally new values with some probability *q*, then

(4) $$p(\theta_{i})=(1-q)p(\theta_{i}|x_{1:i-1})+q(U(\theta ))$$…

where *p*(θ_*i*_|*x*_1:*i* − 1_) is the probability that the parameters θ_*i*_ took some values given all previous observations *x*_1:*i* − 1_, and *U*(θ) is a uniform probability distribution over all possible new values of θ, if there had been a change point.

Bayes' rule expresses a general concept about how an observer's beliefs should be updated in light of new observations (for example, whether observations indicate a change in the underlying environment); it expresses the idea that the degree to which the observer should change his beliefs depends on both the likelihood that previously established parameters are still in force, and the transition function or change-point probability. Hence Bayes' rule captures the two considerations we have argued are important for algorithms that respond adaptably to the rate of change of the environment.

Because these considerations relate so closely to Bayes' theorem, it could be argued that any change-detection model that considers the likelihood that old parameters are still in force, and the prior probability of different parameter values (for example based on a transition function) is Bayesian in nature.

## Uncertainty and learning

In this review we are interested in how learning algorithms adapt to change. A key concept in relation to learning and change is uncertainty. There is a natural relationship between uncertainty and learning in that it is generally true that the purpose of learning is to reduce uncertainty, and conversely, the level of uncertainty about the environment determines how much can be learned (Pearce and Hall, 1980; Dayan and Long, 1998; Dayan et al., 2000). We will now see that two types of uncertainty, *expected uncertainty* and *estimation uncertainty*, which can be loosely related to the concepts of likelihood and transition function just discussed, play different roles in learning and may have distinct neural representations.

### Types of uncertainty

Uncertainty can be divided into two constructs—*risk* or expected uncertainty, and *ambiguity* or estimation uncertainty (Knight, 1921; Dayan and Long, 1998; Courville et al., 2006; Preuschoff and Bossaerts, 2007; Payzan-Lenestour and Bossaerts, 2011).

*Risk* or *expected uncertainty* refers to the uncertainty which arises from the stochasticity inherent in the environment—for example, even if an observer knew with certainty that observations were drawn from some Gaussian distribution *x* ~ 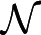 (μ, σ^2^), with known parameter values (known values μ, σ^2^), he would still not be able to predict with certainty the value of the next observation *x*_*i*+1_—because observations are drawn stochastically from a (known) distribution with some variance, σ^2^. Thus, σ^2^ determines the level of expected uncertainty in this environment.

In contrast, uncertainty that arises from the observer's incomplete knowledge of the environment—in our Gaussian example, uncertainty about the values of μ, σ^2^ themselves—is called *estimation uncertainty* or ambiguity (Knight, 1921). Estimation uncertainty is the type of uncertainty that may be reduced by obtaining information, e.g., by increasing the number of observations of the environment. Estimation uncertainty generally increases when the environment is thought to have changed to a new state (since relatively few observations of the new state are available).

Expected uncertainty and estimation uncertainty relate to the two factors we previously discussed in relation to change detection: the likelihood that the same state of the environment is in force now as previously, and the a-priori probability that the state of the environment is not what the observer had previously thought (determined in part by the transition function).

**Figure 2:**
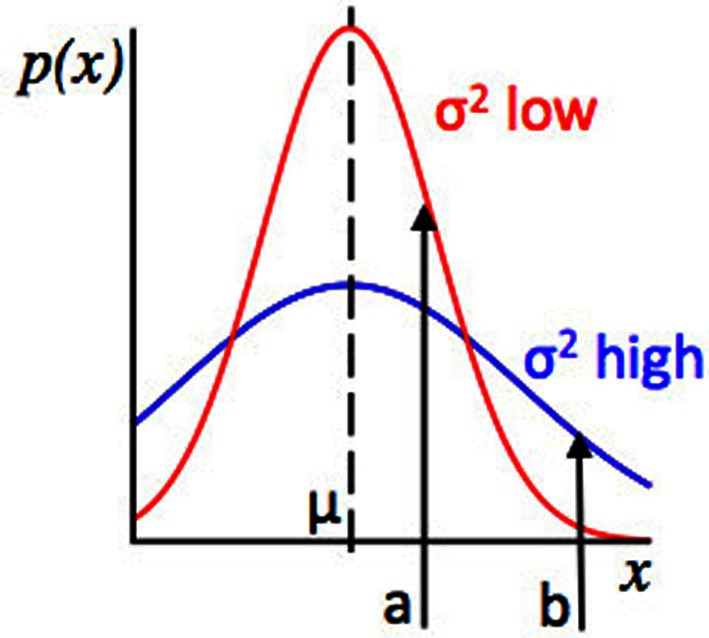
**Relationship between the concepts of Expected Uncertainty and Likelihood**. Plot of values of some observed variable *x* against their probability, given two Gaussian distributions with the same mean. The red distribution has a lower variance, and hence lower expected uncertainty, than the blue distribution. Points *a* and *b* represent possible observed values of *x*. For the red and blue distributions, the distance from the mean (*a* − μ) is the same, but at *a*, the red distribution has higher likelihood (because point *a* has a higher probability under the red distribution than the blue distribution) whilst at point *b*, the blue distribution has a higher likelihood. Consider an algorithm assessing evidence that the environment has changed. If a datapoint *x* = *b* is observed, whether the algorithm infers that there has been a change will depend on the variance or expected uncertainty of the putative pre-change distribution. If the algorithm “thinks” that the red distribution is in force, an observation *x* = *b* is relatively strong evidence for a change in the environment (as *b* is unlikely under the red distribution) but if the algorithm “thinks” the blue distribution is in force, the evidence for change is much weaker, since point *b* is not so unlikely under the blue distribution as it is under the red distribution.

Expected uncertainty affects inferences about the likelihood that the same state of the environment is in force now as previously, because given some observation *x*_*i*_, the strength of evidence for a change in the environment depends not only on how far *x*_*i*_ falls from the expected value E(*x*) but also on the estimated variance of the distribution from which *x* is drawn. For example, in our Gaussian learning model, for some putative μ, the probability of an observation *x*_*i*_ and hence the likelihood of that model parameters μ, σ^2^ take a given value depends both the distance of the observation from the putative model mean, *x*_*i*_ − μ, and on the level of expected uncertainty within the environment, σ^2^: if expected uncertainty (σ^2^) is low, then a given value of (*x*_*i*_ − μ) represents stronger evidence against μ, σ^2^ still being in force, compared to if expected uncertainty (σ^2^) was high. This concept is illustrated in Figure 2.

**Figure 3:**
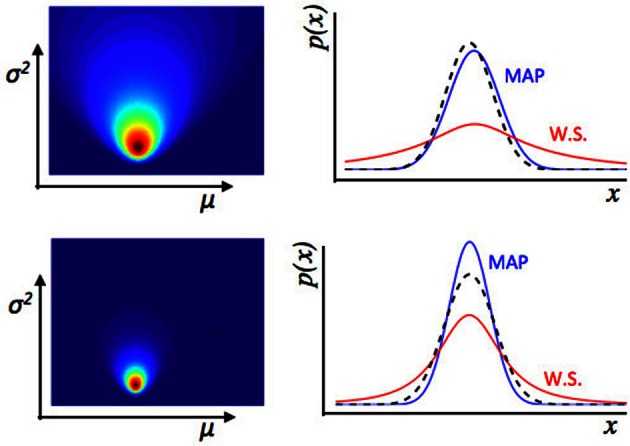
**Illustration of estimation uncertainty**. These plots show the output of a numerical Bayesian estimation of the parameters of a Gaussian distribution. If *x* ~ 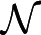 (μ, σ^2^), and some values of *x* are observed, the likelihood of different values for μ, σ^2^ can be calculated jointly using Bayes' rule. The colored plots (left) show the joint likelihood for different pairs of values μ, σ^2^, where each point on the colored image is a possible pair of values μ, σ^2^, and the color represents the likelihood of that pair of values. The line plots (**Right panel**) show the distribution across *x* implied by different values of μ, σ^2^. The dashed black line is the true distribution from which data were drawn. The blue line is the maximum a-posteriori distribution—a Gaussian distribution with values of μ, σ^2^ taken from the peak of the joint distribution over μ, σ^2^ shown on the left. The red line represents a weighted sum (W.S.) of the Gaussian distributions represented by all possible values of μ, σ^2^, weighted by their joint likelihood as shown in the figure to the left. The top represents an estimate of the environment based on fewer data points than the bottom row. With relatively few data points, there is a lot of uncertainty about the values of μ, σ^2^, i.e., estimation uncertainty—illustrated by the broader distribution of likelihood over different possible values of μ, σ^2^ (**Left panel**) in the top than bottom row. Whilst the maximum a-posteriori distribution is a good fit to the “true” distribution from which data were drawn in both cases, if we look at the weighted sum of all distributions, there is a lot more uncertainty for the top row case, based on fewer data points. Hence if the observer uses a weighted sum of all possible values of μ, σ^2^ of the environment to calculate a probability distribution over *x*, the variance of that distribution depends on the level of estimation uncertainty.

**Figure 4:**
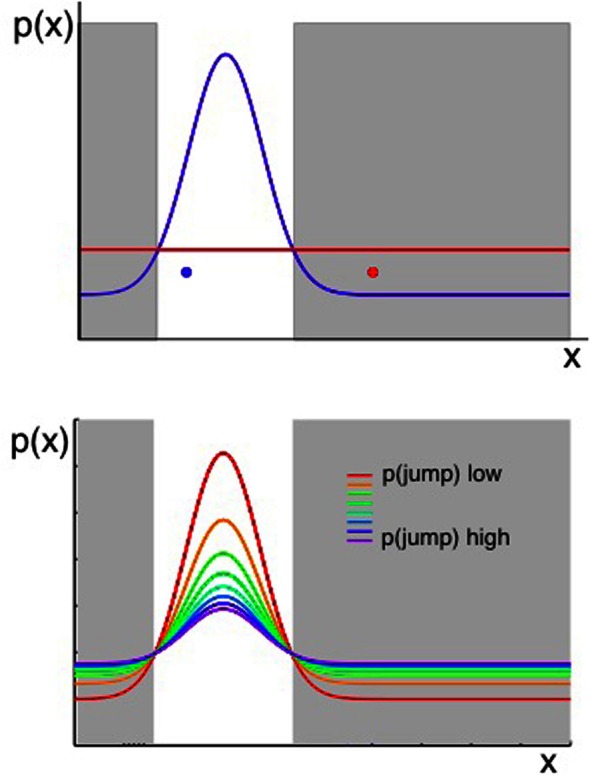
**Two considerations for evaluating whether a change has occurred**. Plots show the probability of observing some value of *x*, given that *x* ~ 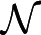 (μ, σ^2^) and the values of μ, σ^2^ can jump to new, unpredicted values as defined in Equation 2. When an observation of the environment is made, an algorithm that aims to determine whether a change has occurred should consider both the likelihood of the previous model of the environment given the new data, and the prior probability of change as determined in part by the transition function. **Top panel:** the probability of an observation taking a value *x* is shown in terms of two distributions. A Gaussian shown in blue represents the probability density across *x* if the most likely state of the environment (the most likely values of μ, σ^2^), given past data, were still in force. The uniform distribution in red represents the probability density across *x* arising from all the possible new states of the environment, if a change occurred. The possible new states are represented by a uniform function (red line in the figure) because, if we consider the probability of each value of *x* under an infinite number of possible states at once (i.e., the value of *x* given each of infinitely many other possible values of μ and σ^2^), the outcome is a uniform distribution over *x*. A change should be inferred if an observation occurs in the gray shaded regions—where the probability of *x* under the uniform (representing change) is higher than the probability under the prior Gaussian distribution. Hence the red data point in Figure 4 should cause the system to infer a change has occurred, whereas the blue data point should not. **Bottom panel:** as above, the probability distribution over *x* is a combination of a Gaussian and a Uniform distribution (representing the most likely parameters of the environment if there has been no change, and the possible new states of the environment if there has been a change, respectively). In this panel, the Gaussian and Uniform components are summed to give a single line representing the distribution over *x*. The different colored lines represent different prior probabilities of change, and hence different relative weightings of the Gaussian and uniform components. Increasing the prior probability of change results in a wider distribution of probability density across all possible values of *x*.

Estimation uncertainty, in contrast, relates more closely to the idea of assessing the a-priori probability of change in the environment. Firstly, the strength of belief in any particular *past state* of the environment affects estimation uncertainty—intuitively, if the observer is not sure about the state of the environment, he may be more willing to adjust his beliefs. Secondly, beliefs about the rate or frequency of change in the environment (i.e., about the transition function) affects estimation uncertainty because if the observer believes the rate of change of the environment to be high, then the extrapolation of past beliefs to predictions about the future state of the environment is more uncertain. These concepts are illustrated in Figures 3, 4.

**Figure 5:**
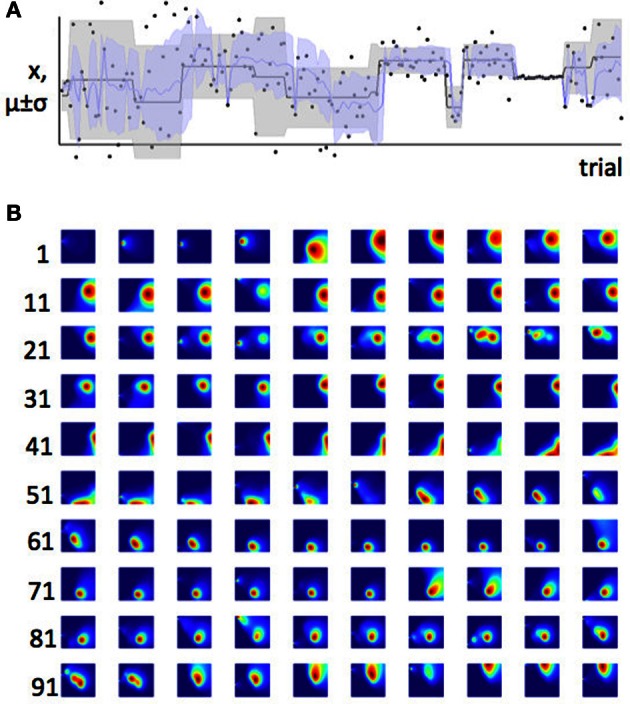
**Bayesian learner estimates the mean and variance of a Gaussian distribution. (A)** Data and maximum likelihood estimates for 200 trials. The actual mean and variance of the distribution from which the data were drawn (generative distribution) are shown in gray. The gray line is the mean and the shaded area is mean ± standard deviation. The model's estimates of these parameters are shown superposed on this, in blue. The actual data point on which the model was trained are shown as black dots. The scale on the y-axis is arbitrary. **(B)** The probability density function across parameter space (for plotting conventions, see Figure 3) for the first 100 trials. Each parameter-space map represents one trial; trials are shown in rows with the first trial number in each row indicated to the left of the row. Possible values of μ_*i*_ are plotted on the y-axis; possible values of σ_*i*_ are plotted on the x-axis. Colors indicate the joint posterior probability for each pair, mu, sigma, after observing data point *x*_*i*_. Increasing values of sigma are plotted from left to right; increasing values of μ_*i*_ are plotted from top to bottom. Hence, for example on trial 10 (top right) the model thinks μ_*i*_ is low, and σ_*i*_ is high. Some interesting sequences of trials are highlighted in Figures 6, 7.

In order to illustrate how the effect of expected and estimation uncertainty on change point detection translate into an influence on learning rate, we can consider a model which observes a series of data points from a Gaussian distribution and uses these sequentially to infer the parameters of that distribution, whilst taking into account the possibility that those parameters have jumped to new values, as in Equation 2. Details of this model are given in the Appendix and its “behaviour” is illustrated in Figure 5.

**Figure 6:**
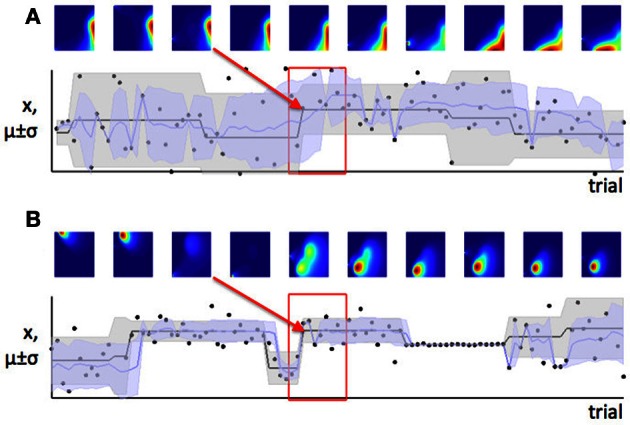
**Learning is faster when expected uncertainty is low**. Panels **(A)** and **(B)** show two sets of trials which include changes of similar magnitude in the mean of the generative distribution (distribution from which data were in fact drawn). In panel **(A)**, the estimate of σ_*i*_ is high (high expected uncertainty) but in panel **(B)**, the estimate of σ_*i*_ is lower—this is indicated by the distribution of probability density from left to right in the colored parameter-space maps, and also the width of the shaded area μ ± σ on the lower plot. The red boxes indicate the set of trials shown in the parameter space maps; the red arrow shows which parameter space map corresponds to the first trial after the change point. Note that the distribution of probability in parameter space changes more slowly when expected uncertainty is high (panel **A**), indicating that learning is slower in this case.

In Figure 2 we saw that when expected uncertainty is high, the deviation of an observed value or set of values from the distribution mean needs to be higher, to offer the same weight of evidence for a change in the underlying model parameters, compared to when expected uncertainty is low. In the case of our Gaussian target locations example, this would mean that when σ^2^ is believed to be high, a given deviation of a sample from the mean (*x* − μ) is weaker evidence for change, compared to when the estimate of σ^2^ is low. In terms of a learning algorithm, this is illustrated in panels **(A)** and **(B)** of Figure 6. Panel **(A)** shows a case where the true mean of the generative distribution changes when σ^2^ is thought to be high (so expected uncertainty is high). Panel **(B)** shows a change of similar magnitude in the generative mean, when σ^2^ is thought to be low. The model adapts much more quickly to the change in the distribution mean in the case with lower expected uncertainty.

**Figure 7:**
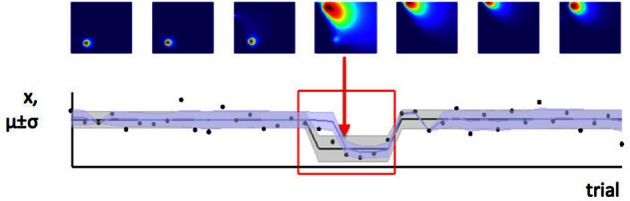
**Change in the environment increases estimation uncertainty**. Here we see a set of trials during which a change point occurs (change point indicated by red arrow). Before the change point, the model has low estimation uncertainty (probability density is very concentrated in a small part of parameter space, as seen from the first three parameter space maps). When the change point is detected, estimation uncertainty increases as the model initially has only one data point on which to base its estimate of the new parameters of the distribution. Over the next few trials, estimation uncertainty decreases (probability density becomes concentrated in a smaller part of parameter space again).

In contrast, we have argued that the level of estimation uncertainty or ambiguity is more closely related to the second consideration, the probability of change itself. Consider the process by which probability densities over the model parameters are updated in our Bayesian learning model. A-priori (before a certain data point *x*_*i*_ is observed), if the probability of change is believed to be high, estimation uncertainty over the parameters μ and σ^2^ is also high—this is the effect illustrated in Figure 6. Conversely, a-posteriori (after a data point or data points are observed), estimation uncertainty is increased if evidence for a change-point is observed (i.e., a data point or set of data points which are relatively unlikely given the putative current state of the environment), (Dayan and Long, 1998; Courville et al., 2006). We can see this in Figure 7. As the model starts to suspect that the parameters of the environment have changed, the spread of probability density across parameter space (i.e., estimation uncertainty) increases. As more data are observed from the new distribution, the estimate of the new parameters of the environment improves, and estimation uncertainty decreases. Hence estimation uncertainty is related to both to the a-priori expectation of change, and the a-posteriori probability that a change may have occurred.

The role of estimation uncertainty in determining how much can be learned can be related to concepts in both Bayesian theory (Behrens et al., 2007) and classic associative learning theory (Pearce and Hall, 1980): in the terminology of classical conditioning, estimation uncertainty can be equated with *associability* (Dayan and Long, 1998; Dayan et al., 2000)—associability being a term in formal learning theory which defines how much can be learned about a given stimulus, where the amount that can be learned is inversely related to how much is already known about the stimulus (Pearce and Hall, 1980). Low estimation uncertainty means low associability—which means minimal learning. Similarly, estimation uncertainty relates to the *learning rate*—α in the Rescorla–Wagner model of reinforcement learning (Rescorla and Wagner, 1972; Behrens et al., 2007)—because higher estimation uncertainty is associated with faster learning.

## Top down control of estimation uncertainty?

In a stable environment, estimation uncertainty—uncertainty about the parameters of the environment—generally decreases over time, as more and more observations are made to be consistent with a particular state of the environment. Indeed it has been argued that the main goal of a self-organizing system like the brain is to reduce surprise by improving the match between its internal representations of the environment and the environment itself (Friston and Kiebel, 2009; Friston, 2010), i.e., to reduce estimation uncertainty as well as estimation error.

Whilst additional observations of the environment tend to decrease estimation uncertainty, estimation uncertainty is driven up by observations that suggest a change may have occurred in the environment: surprising stimuli are associated with increases in the learning rate (Courville et al., 2006). We might think of this as bottom-up or data-driven control of the level of estimation uncertainty in the model, or equivalently the learning rate, or the prior expectation of change.

However, it is also possible to imagine situations in which it might be advantageous to control estimation uncertainty (or the learning rate) top down instead of bottom up—i.e., to *actively* increase the learning rate in order to “make space” for new information about the environment. One such situation would be when an observer is actively exploring his environment and hence presumably wishes to adapt his internal model of the environment to take into account the new information obtained by exploring. Indeed, change of context (moving an animal from one location to another) is associated with increased learning rate in experimental animals (Lovibond et al., 1984; Hall and Channell, 1985; McLaren et al., 1994).

## Neural representations of estimation uncertainty and learning rate

A common set of neural phenomena are associated with the rate of learning, processing of stimuli that could indicate a change in the environment, and active exploration of the environment; these phenomena could be conceptualized computationally in terms of control of the level of estimation uncertainty in the brain's models of the environment.

Neuroanatomically, an area of particular interest in relation to estimation uncertainty is the anterior cingulate cortex (ACC). Activity in the ACC has been shown to correlate with learning rate such that, in environments in which the environment changes frequently and observers learn quickly about change (i.e., conditions of high estimation uncertainty), the ACC is more active (Behrens et al., 2007). The ACC is also activated when people receive feedback about their actions or beliefs that causes them to modify their behavior on future trials (and by implication, to modify their internal model of the environment) (Debener et al., 2005; Cohen and Ranganath, 2007; Matsumoto et al., 2007)—this activity, which has been observed using fMRI and electrophysiological recordings, is probably the source of the error- or feedback-related negativity (ERN; Debener et al., 2005).

Interestingly, ACC activity may be more closely related to the *forgetting* of old beliefs about the environment (and hence the increasing of estimation uncertainty), than to new learning. In a particularly relevant study Karlsson et al. (2012), showed that in rats performing a two-alternative probabilistic learning task, patterns of activity in the ACC underwent a major change in activity when the probabilities associated with each of the two options reversed. Importantly, rats' behavior around a probability reversal (when the values associated with each lever switched) had three distinct phases—before the reversal, rats showed a clear preference for the high value lever, but when the probabilities reversed there was a period in which the rats showed no preference for either lever (they probed each lever several times as if working out the new values associated with each lever) before settling down into a new pattern of behavior that favored the new high value lever. The ACC effect was associated with the point at which rats abandoned their old beliefs about the environment in favor of exploration and the acquisition of new information (and hence, should have had raised levels of estimation uncertainty)—rather than at the time at which a new model of the environment started to govern behavior.

Further experiments have reported ACC activity when participants make the decision to explore their environment rather than to exploit known sources of reward (Quilodran et al., 2008), or to forage for new reward options rather than choosing between those options immediately available to them (Kolling et al., 2012)—again, these are cases in which estimation uncertainty in the brain's internal models could be actively raised, to facilitate the acceptance of new information in the new environment (Dayan, 2012).

Neurochemically, Dayan and colleagues have proposed that the neuromodulator noradrenaline (also called norepinephrine) signals estimation uncertainty. Evidence from pupilometry studies suggests that noradrenaline levels [which are correlated with pupil dilation (Aston-Jones and Cohen, 2005)] are high when estimation uncertainty is high in a gambling task (Preuschoff et al., 2011). Increases in pupil dilation have been demonstrated both circumstances that should drive estimation uncertainty bottom-up [when data are observed that suggest a change point has occurred (Nassar et al., 2012)], and top down [during exploratory behavior (Nieuwenhuis et al., 2005)].

Pupil diameter is increased in conditions when observers think the rate of change in the environment is high, and is phasically increased when observers detect a change in the environment (Nassar et al., 2012). Hence tonic noradrenaline levels could be said to represent the prior probability of change in the environment, whilst phasic noradrenaline may represent a-posteriori evidence (based on sensory input) that a change is occurring or has occurred at a given time point (Bouret and Sara, 2005; Dayan and Yu, 2006; Sara, 2009).

Interestingly, whilst events which are surprising in relation to a behaviorally-relevant model of the environment are associated with an increase in noradrenaline release [29,30] and pupil diameter [31], it has also been shown that irrelevant surprising events which cause an increase in pupil diameter also cause an increase in learning rate (Nassar et al., 2012) suggesting a rather generalized mechanism by which the malleability of neural circuits may be affected by surprise, in accordance with behavioral evidence that surprising events affect the learning rate (Courville et al., 2006).

The mechanism by which noradrenaline represents or controls estimation uncertainty is not known, although two appealing theoretical models are that noradrenaline acts on neural models of the environment by adjusting the gain function of neurons (Aston-Jones and Cohen, 2005), or by acting as a “reset” signal that replaces old models of the environment with uninformative distributions, to make space for new learning (Bouret and Sara, 2005; Sara, 2009).

The involvement of the ACC and noradrenaline in the control/representation of estimation uncertainty may be linked, because the ACC has strong projections to the nucleus that produces noradrenaline, the locus coeruleus (Sara and Herve-Minvielle, 1995; Jodo et al., 1998).

Whilst there is currently little consensus on the representation of learning rate and uncertainty in the brain, the data reviewed here do begin to suggest a mechanism by which estimation uncertainty and learning rate are controlled neurally, which is involved both when uncertainty/learning is driven bottom-up (by observations that suggest the environment is changing) and when they are driven top-down (such as when agents actively quit a familiar environment and explore a novel one).

### Conflict of interest statement

The author declares that the research was conducted in the absence of any commercial or financial relationships that could be construed as a potential conflict of interest.

## Appendix

### Learning model for Figures 4–7

Let data *x* be drawn from a Gaussian distribution with unknown mean μ and variance σ^2^. The values of μ and σ^2^ occasionally jump to new values; the probability of such a jump occurring between and pair of observations is fixed at some value *q*. For simplicity in this example we assume *q* is known, but it is also possible to infer *q* from the data (Nassar et al., 2010; Wilson et al., 2010).

Then the structure of the environment can be described as follows:

(6) $$\mu_{i},\sigma_{i}^{2}=\;\{\begin{array}{ll} \mu_{i-1},\sigma_{i-1}^{2}\; & \text{if }J=0\\ {\text{U}}^{2}\text{​}\left(\mu_{\text{min}},\mu_{\text{max},}\sigma^{2}{}_{\text{min},}\sigma^{2}{}_{\text{max}}\right)\; & \text{if }J=1 \end{array}\;$$

where *J* is a binary variable determining the probability of a jump, such that *J* follows a Bernouilli with probability *q*.

(7) J~B(q)

Then the values for μ_*i*_ and σ_*i*_ can be inferred from the data using Bayes' rule as follows:

(8) $$p(\mu_{i},\sigma_{i}^{2}|x_{1\;:\;i})=p(x_{i}|\mu_{i},\sigma_{i}^{2})p(\mu_{i},\sigma_{i}^{2}|x_{1:i-1})$$

where the likelihood is

… and the prior is derived from the posterior on the previous trial, incorporating a uniform “leak” over parameter space to represent the possibility that the values of the parameters have changed since the previous observation:

(10) $$\begin{array}{l} p(\mu_{i},\sigma_{i}^{2}|x_{1:i-1})=(1-q)p(\mu_{i-1},\sigma_{i-1})\\ \;+q\text{​}\left({\text{U}}^{2}\text{​}\left(\mu_{\text{min}},\mu_{\text{max},}\sigma^{2}{}_{\text{min},}\sigma^{2}{}_{\text{max}}\right)\right) \end{array}$$

On trial 1, the prior over parameter space is uniform.

## References

[B1] Aston-Jones G. Cohen J. D. (2005). An integrative theory of locus coeruleus-norepinephrine function: adaptive gain and optimal performance. Annu. Rev. Neurosci. 28, 403–450 10.1146/annurev.neuro.28.061604.135709 16022602

[B2] Behrens T. E. Woolrich M. W. Walton M. E. Rushworth M. F. (2007). Learning the value of information in an uncertain world. Nat. Neurosci. 10, 1214–1221 10.1038/nn1954 17676057

[B3] Bogacz R. Brown E. Moehlis J. Holmes P. Cohen J. D. (2006). The physics of optimal decision making: a formal analysis of models of performance in two-alternative forced-choice tasks. Psychol. Rev. 113, 700–765 10.1037/0033-295X.113.4.700 17014301

[B4] Bouret S. Sara S. J. (2005). Network reset: a simplified overarching theory of locus coeruleus noradrenaline function. Trends Neurosci. 28, 574–582 10.1016/j.tins.2005.09.002 16165227

[B5] Cohen M. X. Ranganath C. (2007). Reinforcement learning signals predict future decisions. J. Neurosci. 27, 371–378 10.1523/JNEUROSCI.4421-06.2007 17215398 PMC6672075

[B6] Courville A. C. Daw N. D. Touretzky D. S. (2006). Bayesian theories of conditioning in a changing world. Trends Cogn. Sci. 10, 294–300 10.1016/j.tics.2006.05.004 16793323

[B7] Dayan P. (2012). Twenty-five lessons from computational neuromodulation. Neuron 76, 240–256 10.1016/j.neuron.2012.09.027 23040818

[B8] Dayan P. Kakade S. Montague P. R. (2000). Learning and selective attention. Nat. Neurosci. 3, 1218–1223 10.1038/81504 11127841

[B9] Dayan P. Long T. (1998). Statistical models of conditioning, in Advances in Neural Information Processing Systems 10, eds Jordan M. I. Kearns M. J. Solla S. A. (Cambridge, MA: MIT Press), 117–123

[B10] Dayan P. Yu A. J. (2006). Phasic norepinephrine: a neural interrupt signal for unexpected events. Network 17, 335–350 10.1080/09548980601004024 17162459

[B11] Debener S. Ullsperger M. Siegel M. Fiehler K. Von Cramon D. Y. Engel A. K. (2005). Trial-by-trial coupling of concurrent electroencephalogram and functional magnetic resonance imaging identifies the dynamics of performance monitoring. J. Neurosci. 25, 11730–11737 10.1523/JNEUROSCI.3286-05.2005 16354931 PMC6726024

[B12] Doya K. (2002). Metalearning and neuromodulation. Neural Netw. 15, 495–506 10.1016/S0893-6080(02)00044-8 12371507

[B13] Friston K. (2010). The free-energy principle: a unified brain theory? Nat. Rev. Neurosci. 11, 127–138 10.1038/nrn2787 20068583

[B14] Friston K. Kiebel S. (2009). Predictive coding under the free-energy principle. Philos. Trans. R. Soc. Lond. B Biol. Sci. 364, 1211–1221 10.1098/rstb.2008.0300 19528002 PMC2666703

[B15] Hall G. Channell S. (1985). Differential-effects of contextual change on latent inhibition and on the habituation of an orienting response. J. Exp. Psychol. Anim. Behav. Process. 11, 470–481 10.1037/0097-7403.11.3.470

[B16] Jodo E. Chiang C. Aston-Jones G. (1998). Potent excitatory influence of prefrontal cortex activity on noradrenergic locus coeruleus neurons. Neuroscience 83, 63–79 10.1016/S0306-4522(97)00372-2 9466399

[B16a] Karlsson M. P. Tervo D. G. Karpova A. Y. (2012). Network resets in medial prefrontal cortex mark the onset of behavioral uncertainty. Science 338, 135–139 10.1126/science.1226518 23042898

[B17] Knight F. H. (1921). Risk, Uncertainty and Profit. Boston MA: Hart, Schaffner and Marx

[B18] Kolling N. Behrens T. E. Mars R. B. Rushworth M. F. (2012). Neural mechanisms of foraging. Science 336, 95–98 10.1126/science.1216930 22491854 PMC3440844

[B19] Lovibond P. F. Preston G. C. Mackintosh N. J. (1984). Context specificity of conditioning, extinction, and latent inhibition. J. Exp. Psychol. Anim. Behav. Process. 10, 360–375 10.1037/0097-7403.10.3.360

[B20] Matsumoto M. Matsumoto K. Abe H. Tanaka K. (2007). Medial prefrontal cell activity signaling prediction errors of action values. Nat. Neurosci. 10, 647–656 10.1038/nn1890 17450137

[B21] McGrayne S. B. (2011). The Theory That Would Not Die: How Bayes' Rule Cracked the Enigma Code, Hunted Down Russian Submarines, and Emerged Triumphant from Two Centuries of Controversy. New Haven, CT: Yale University Press

[B22] McLaren I. P. L. Bennett C. Plaisted K. Aitken M. Mackintosh N. J. (1994). Latent inhibition, context specificity, and context familiarity. Q. J. Exp. Psychol. B 47, 387–400 10.1080/14640749408401366 7809404

[B23] Nassar M. R. Rumsey K. M. Wilson R. C. Parikh K. Heasly B. Gold J. I. (2012). Rational regulation of learning dynamics by pupil-linked arousal systems. Nat. Neurosci. 15, 1040–1046 10.1038/nn.3130 22660479 PMC3386464

[B24] Nassar M. R. Wilson R. C. Heasly B. Gold J. I. (2010). An approximately Bayesian delta-rule model explains the dynamics of belief updating in a changing environment. J. Neurosci. 30, 12366–12378 10.1523/JNEUROSCI.0822-10.2010 20844132 PMC2945906

[B25] Nieuwenhuis S. Aston-Jones G. Cohen J. D. (2005). Decision making, the P3, and the locus coeruleus-norepinephrine system. Psychol. Bull. 131, 510–532 10.1037/0033-2909.131.4.510 16060800

[B26] Payzan-Lenestour E. Bossaerts P. (2011). Risk, unexpected uncertainty, and estimation uncertainty: Bayesian learning in unstable settings. PLoS Comput. Biol. 7:e1001048 10.1371/journal.pcbi.1001048 21283774 PMC3024253

[B27] Pearce J. M. Hall G. (1980). A model for pavlovian learning—variations in the effectiveness of conditioned but not of unconditioned stimuli. Psychol. Rev. 87, 532–552 10.1037/0033-295X.87.6.532 7443916

[B28] Preuschoff K. Bossaerts P. (2007). Adding prediction risk to the theory of reward learning. Ann. N.Y. Acad. Sci. 1104, 135–146 10.1196/annals.1390.005 17344526

[B29] Preuschoff K., T Hart B. M. Einhauser W. (2011). Pupil dilation signals surprise: evidence for noradrenaline's role in decision making. Front. Neurosci. 5:115 10.3389/fnins.2011.00115 21994487 PMC3183372

[B30] Quilodran R. Rothe M. Procyk E. (2008). Behavioral shifts and action valuation in the anterior cingulate cortex. Neuron 57, 314–325 10.1016/j.neuron.2007.11.031 18215627

[B31] Rescorla R. A. Wagner A. R. (1972). A theory of Pavlovian conditioning: variations in the effectiveness of reinforcement and nonreinforcement, in Classical Conditioning II: Current Research and Theory, eds Black A. H. Prokasy W. F. (New York, NY: Appleton-Century Crofts), 64–99 10.1037/a0030892

[B32] Sara S. J. (2009). The locus coeruleus and noradrenergic modulation of cognition. Nat. Rev. Neurosci. 10, 211–223 10.1038/nrn2573 19190638

[B33] Sara S. J. Herve-Minvielle A. (1995). Inhibitory influence of frontal cortex on locus coeruleus neurons. Proc. Natl. Acad. Sci. U.S.A. 92, 6032–6036 10.1073/pnas.92.13.6032 7597075 PMC41636

[B34] Usher M. McClelland J. L. (2001). The time course of perceptual choice: the leaky, competing accumulator model. Psychol. Rev. 108, 550–592 10.1037/0033-295X.108.3.550 11488378

[B35] Wilson R. C. Niv Y. (2011). Inferring relevance in a changing world. Front. Hum. Neurosci. 5:189 10.3389/fnhum.2011.00189 22291631 PMC3264902

[B36] Wilson R. C. Nassar M. R. Gold J. I. (2010). Bayesian online learning of the hazard rate in change-point problems. Neural Comput. 22, 2452–2476 10.1162/NECO_a_00007 20569174 PMC2966286

